# Selenium Biofortification in Radish Enhances Nutritional Quality via Accumulation of Methyl-Selenocysteine and Promotion of Transcripts and Metabolites Related to Glucosinolates, Phenolics, and Amino Acids

**DOI:** 10.3389/fpls.2016.01371

**Published:** 2016-09-14

**Authors:** Michela Schiavon, Chiara Berto, Mario Malagoli, Annarita Trentin, Paolo Sambo, Stefano Dall'Acqua, Elizabeth A. H. Pilon-Smits

**Affiliations:** ^1^Department of Agronomy, Food, Natural Resources, Animals and the Environment, University of PadovaLegnaro, Italy; ^2^Biology Department, Colorado State UniversityFort Collins, MS, USA; ^3^Department of Pharmaceutical and Pharmacological Sciences, University of PadovaPadova, Italy

**Keywords:** selenium, fortification technologies, radish (*Raphanus sativus* L.), nutritional quality enhancement, glucosinolates

## Abstract

Two selenium (Se) fertilization methods were tested for their effects on levels of anticarcinogenic selenocompounds in radish (*Raphanus sativus*), as well as other nutraceuticals. First, radish was grown on soil and foliar selenate applied 7 days before harvest at 0, 5, 10, and 20 mg Se per plant. Selenium levels were up to 1200 mg Se/kg DW in leaves and 120 mg Se/kg DW in roots. The thiols cysteine and glutathione were present at 2–3-fold higher levels in roots of Se treated plants, and total glucosinolate levels were 35% higher, due to increases in glucoraphanin. The only seleno-aminoacid detected in Se treated plants was Se-methyl-SeCys (100 mg/kg FW in leaves, 33 mg/kg FW in roots). The levels of phenolic aminoacids increased with selenate treatment, as did root total nitrogen and protein content, while the level of several polyphenols decreased. Second, radish was grown in hydroponics and supplied with 0, 5, 10, 20, or 40 μM selenate for 1 week. Selenate treatment led to a 20–30% increase in biomass. Selenium concentration was 242 mg Se/kg DW in leaves and 85 mg Se/kg DW in roots. Cysteine levels decreased with Se in leaves but increased in roots; glutatione levels decreased in both. Total glucosinolate levels in leaves decreased with Se treatment due to repression of genes involved in glucosinolates metabolism. Se-methyl-SeCys concentration ranged from 7–15 mg/kg FW. Aminoacid concentration increased with Se treatment in leaves but decreased in roots. Roots of Se treated plants contained elevated transcript levels of sulfate transporters (Sultr) and ATP sulfurylase, a key enzyme of S/Se assimilation. No effects on polyphenols were observed. In conclusion, Se biofortification of radish roots may be achieved via foliar spray or hydroponic supply. One to ten radishes could fulfill the daily human requirement (70 μg) after a single foliar spray of 5 mg selenate per plant or 1 week of 5–10 μM selenate supply in hydroponics. The radishes metabolized selenate to the anticarcinogenic compound Se-methyl-selenocysteine. Selenate treatment enhanced levels of other nutraceuticals in radish roots, including glucoraphanin. Therefore, Se biofortification can produce plants with superior health benefits.

## Introduction

Worldwide, interest in the biological impact of selenium (Se) on food quality is increasing, as this element is an essential micronutrient for humans and animals. Selenium deficiency occurs in several countries, especially where Se concentration in soil and food crops is very low (Broadley et al., 2006). It is estimated that between 0.5 and 1 billion people suffer from Se deficiency (Combs, 2001) because their Se consumption is lower than the recommended dietary allowance (RDA) of 50–70 μg Se day^−1^ (USDA, 2012). Low Se intake may result in several health disorders including heart disease, reduced fertility, hypothyroidism, oxidative stress-related conditions, and weakened immune system (Rayman, 2012). Conversely, an adequate dietary Se supplement confers a variety of health benefits, since Se is present as selenocysteine (SeCys) in at least 25 different proteins, among which are the powerful antioxidant selenoglutathione peroxidases (Rayman, 2009). Organic selenocompounds such as selenomethionine (SeMet), methylselenocysteine (MSeC), and methylselenol display anticarcinogenic properties (Medina et al., 2001; Combs, 2005; Vinceti et al., 2014; Fernandes and Gandin, 2015). On the other end of the spectrum, Se at high dosages may also be harmful to humans and animals, due to the capacity of inorganic forms of Se to cause oxidative stress and of SeCys to replace Cys in proteins (Wilber, 1980; Misra et al., 2015). Excess Se intake has, among other things, been shown to increase the risk of type-2 diabetes in humans (Rayman, 2012; Roman et al., 2014).

Selenium has not been recognized as essential to higher plants, but is considered a beneficial element for plants (Pilon-Smits et al., 2009). Since plants represent the major dietary soured of Se to human and animal consumption worldwide, studies on plant Se accumulation and metabolism do not only have intrinsic merit, but are relevant to human and animal nutrition. Due to its chemical similarity to sulfur (S), Se is taken up and assimilated by plants principally via S transporters and enzymes. The main bioavailable form of Se in soils is selenate, which is taken up by root sulfate transporters (White et al., 2004; El Kassis et al., 2007). The existence of a common mechanism for the uptake of selenate and sulfate in plants was first established in *Arabidopsis thaliana* mutants lacking the functional high-affinity root sulfate transporter SULTR1;2 (Shibagaki et al., 2002). Because the mutation conferred to these plants elevated resistance to selenate, SULTR1;2 has been identified as the main transporter involved in selenate influx into the plant roots.

The low affinity sulfate transporter SULTR2;1, which is expressed in the xylem and phloem parenchyma cells of leaves and xylem parenchyma and pericycle cells of roots in *Arabidopsis*, is involved in the uptake of sulfate from the apoplast within the vascular bundle (Hawkesford, 2003) and has been suggested to function in the root-to-shoot transport of sulfate in *Arabidopsis*, in synergy with SULTR3;5 (Takahashi et al., 1997, 2000; Kataoka et al., 2004).

Once transported to the shoot, inside leaf cells selenate is then activated by the enzyme ATP sulphurylase (ATPS), forming adenosine 5′-phosphoselenate (APSe). The ATPS gene family in *A. thaliana* includes four members: ATPS1 (Leustek et al., 1994), ATPS2, ATPS3 (Murillo and Leustek, 1995), and ATPS4 (Hatzfeld et al., 2000). Overexpression of ATPS1 in *Brassica juncea* resulted in enhanced Se assimilation and accumulation, suggesting that the activation of selenate to APSe represents one of the rate-limiting steps for selenate assimilation in plants (Pilon-Smits et al., 1999). After activation, selenate is reduced via selenite to selenide and incorporated into selenocysteine (SeCys) and selenomethionine (SeMet) (Sors et al., 2005). The non-specific insertion of these seleno-amino acids (particularly SeCys) in the molecular structure of proteins is thought to result in the loss of their correct folding and function (Van Hoewyk, 2013).

Selenium-rich plants may have potential as fortified food with enhanced nutritional quality (Zhu et al., 2009). For instance, some species belonging to the *Brassicaceae* (Crucifer) family can accumulate Se up to 0.1–1.5% in plant dry weight and may contain high levels of methyl-SeCys, which has been reported to have anticarcinogenic properties (Freeman et al., 2006; Pilon-Smits and LeDuc, 2009; White, 2016). An additional advantage of the consumption of vegetables from this family is that they have been strongly associated with decreased cancer risk because they are rich in bioactive compounds with chemoprotective and antioxidant properties, such as glucosinolates and phenolics (Wagner et al., 2013).

Glucosinolates (GLSs) are nitrogen (N)- and sulfur-containing glycosides derived from a variety of amino acids and are responsible for the sharp taste of cruciferous vegetables (Matich et al., 2012). They are produced by the plant as part of a defense mechanism against insect and herbivore predators (Jørgensen et al., 2015). GLSs can be clustered into three major structural groups on the basis of the amino acid precursor of the side chain: indole glucosinolates derived from tryptophan, aliphatic glucosinolates derived from methionine, and aromatic glucosinolates derived from phenylalanine or tyrosine (Agerbirk and Olsen, 2012). Within plant cells, GLSs can be hydrolyzed by the enzyme myrosinase upon plant injury or during food processing, thus leading to the formation of biologically active compounds, like indoles and isothiocyanates (ITC), which serve as cancer-preventive agents in mammals (Dinkova-Kostova, 2013).

The application of Se-containing fertilizers to plants is one of the possible strategies to obtain plants fortified with Se. However, when Se fertilization is performed via selenate supplementation to cruciferous vegetables, Se may interfere with cysteine and methionine biosynthesis and potentially exert adverse effects on glucosinolate accumulation, as selenate and sulfate share the same assimilation pathway. In the case of Se-enriched broccoli, contrasting results have been reported so far (Robbins et al., 2005; Hsu et al., 2011). Selenium fertilization could also compromise the accumulation of other bioactive components like phenolic acids in these vegetables (Robbins et al., 2005), or increase their content at low selenate dosages in tomato plants (Schiavon et al., 2013). Se can also hamper molybdenum (Mo) uptake by plants (Harris et al., 2014), and thus decrease the activity of the enzyme nitrate reductase, which needs Mo as a cofactor. As a result, the nitrogen assimilation pathway may be affected by Se.

Factors such as cultivation methods, Se dosage, selenate/sulfate ratio in the growth medium, and duration of Se fertilization may be important key determinants for the effects of Se on S, N and phenol metabolic routes. On this account, in the present study two different Se fertilization methods (foliar Se application to plants cultivated in soil and Se supplementation in the nutrient solution to plants grown in hydroponics) were tested to determine the most appropriate method to obtain radish (*Raphanus sativus L.*) plants biofortified with Se without negative effects on the content of beneficial phytochemicals in edible tissues. The tap root of this plant species is the main edible organ, but leaves, seeds, and flowers can also be consumed. About seven million tons of radishes are produced yearly (about 2% of global vegetable production), and these plants are rich in potassium, zinc, glucosinolates, and antioxidants like Vitamins C and B, flavonoids and anthocyanins (Schippers, 2004).

Given the high GLS content in radishes, to better describe the potential impact of Se fertilization on glucosinolate production, the effect of selenate application on sulfur nutrition in radish was investigated at the molecular level. Specifically, the expression was assayed of genes encoding sulfate transporters, ATP sulfurylase, or involved in GLS biosynthesis and breakdown. In addition, tissue levels of Se and of beneficial nutraceuticals were determined.

## Materials and methods

### Experimental design and plant growth

#### Soil experiment

**Figure 1:**
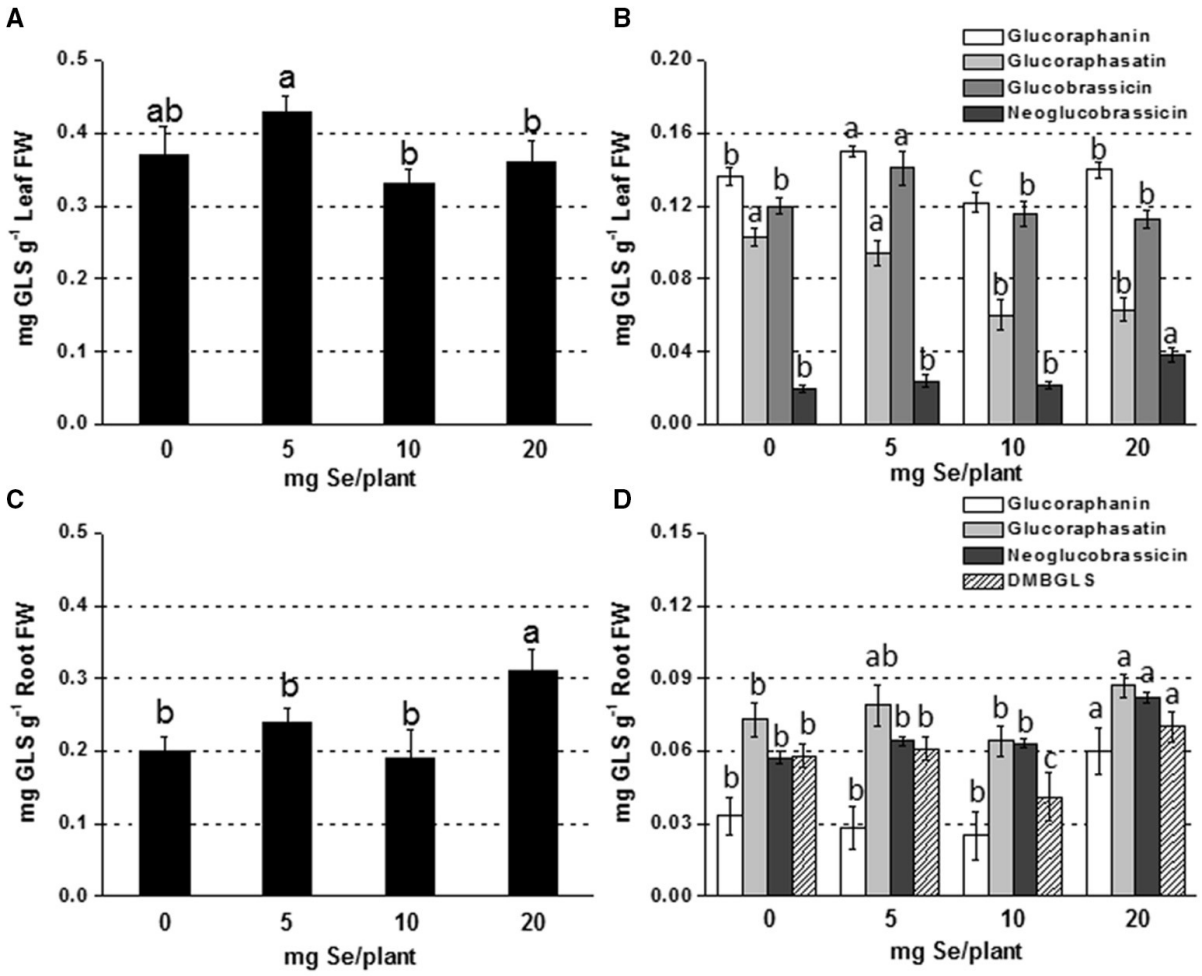
**Concentration of total (A,C) and individual (B,D) glucosinolates (GLSs) in radish leaves (A,B) and roots (C,D) grown in soil and sprayed with 0, 5, 10, or 20 mg Se per plant**. Letters above bars indicate significant differences between the means (*n* = 3, ±*SD*, *p* < 0.05). In **(B,D)** the statistical analysis was performed between values of GLS concentration vs. Se concentration for each individual GLS.

For the soil experiment, seeds of radish (*Raphanus sativus L.*, cv. Saxa) were allowed to germinate in vermiculite for 10 days inside a greenhouse under natural light conditions (April-May, average day/night temperature 18/15°C and photoperiod 14/10 h). Germinated seedlings were then transferred to 1.5 kg-pots (two plants per pot) containing peat, soil and perlite in the ratio 60:30:10. Pots were divided over four groups (5 pot per group, *n* = 5) containing 10 plants each (Figure 1SA), and were watered twice a day.

After 1 month, when the red root was well developed (the edible part of the plant), a unique foliar application of selenate (Na_2_SeO_4_) to three of the plant groups was performed at dosages of 5, 10, or 20 mg per plant. One group of plants was sprayed with an equal volume of water and served as control. During foliar Se treatment, the soil around the plants were covered in order to avoid Se contamination of soil. One week after Se application, plants were harvested, carefully washed with distilled water, divided into leaves and roots and weighed separately. Part of leaves and roots were dried for 2 days at 70°C for the measurement of dry weight, as well as for determination of elemental and anion content. The remaining plant material (leaves and roots) was kept at −80°C for further analyses and used for amino acid, polyphenol, protein, and GLS determination. The experimental design for seedling growth was randomized (the pots were re-arranged three times a week) and the entire experiment was replicated two times.

#### Hydroponic experiment

Seeds of radish (*Raphanus sativus L.*, cv. Saxa) were surface-sterilized by rinsing in 70% (v/v) ethanol for 30–60 s, then in 5% (v/v) sodium hypochlorite (NaClO) for 30 min while rocking on a platform, and washed in distilled water for 5 × 10 min. The seeds were allowed to germinate and grow for 8 days in half-strength Murashige and Skoog (MS) agar medium (Murashige and Skoog, 1962) inside a chamber with a 14 h light/10 h dark cycle, air temperature of 26/21°C, relative humidity of 70/85% and at a photon flux density (PFD) of 280 mol m^−2^s^−1^.

Germinated seedlings were transferred to polystyrene containers (48 cm × 32 cm × 6 cm) filled with vermiculite (40 plants per plateau). Each container was floated in one 40 L tank containing a thoroughly aerated nutrient solution with the following composition (mM): KH_2_PO_4_ (0.63), Ca(NO_3_)_2_ (2), KNO_3_ (3), MgSO_4_ (1.5), FeNaEDTA (0.040), plus micronutrients. The nutrient solution was renewed every 6 days. At 30 days since the transplant, Se in the form of selenate (Na_2_SeO_4_) was added to the nutrient solution in the form of a unique application at the following concentrations: 5, 10, 20, or 40 μM (Figure 1SB). This corresponded with 0.4, 0.8, 1.2, and 1.6 mg Se per plant, respectively (Table 6S). A group of plants was not exposed to selenate and served as the control.

All plants grown under hydroponic conditions developed the red root and some fine white roots, which were both used for elemental quantification and growth measurement. The white roots were also used for the gene expression analysis, while the red roots were used for all the remaining determinations.

One week after the beginning of the Se treatment, plants were harvested, carefully washed with distilled water and dried with blotting paper. Leaves and roots (white and red) of a number of plants was immediately frozen with liquid nitrogen and kept at −80°C for further analyses. For fresh weight measurement, 20 plants per treatment were divided into red roots, white roots and shoot, and weighed separately. Samples were next placed in a drying oven for 2 days at 70°C for the measurement of dry weight.

The experimental design for plant growth was randomized (the trays were re-arranged twice a week) and the entire experiment was replicated two times (each with 20 replicates per treatment).

### Determination of total Se, macro- and microelements

Foliar and root tissues of radish plants were dried for 48 h at 80°C and then digested in nitric acid as described by Zarcinas et al. (1987). Inductively coupled plasma atomic emission spectroscopy (ICP-AES) was used as described by Fassel (1978) to determine each digest's elemental concentrations (Se, S, Mo, Mn, Mg, Ca, Fe, Cu). Quantification of C and N was performed using an elemental analyzer (Vario MACRO CNS, Hanau, Germany).

### Analysis of sulfate and nitrate content

Dry foliar and root tissues (200 mg) were ground in liquid nitrogen and then 10 mL of distilled water were added. The samples were incubated for 2 h in a heating block at 85°C. The obtained extracts were filtered (0.45 μm, Millipore) and analyzed for anion concentration by HPLC using a Dionex IonPac AS11 4 mm column, coupled to a guard column AG 14 and a CD20 Conductivity Detector. The column was eluted over a period of 18 min with 3.5 mM Na_2_CO_3_/1 mM NaHCO_3_ in H_2_O, at a flow rate of 0.9 mL min^−1^ and at 1400 PSI pressure.

### Determination of low molecular weight thiol compounds

Frozen leaf samples (250 mg) from five biological replicates were ground with a mortar and pestle to extract soluble antioxidants with 0.1 N HCl and 1 mM EDTA. Following centrifugation at 10,000 g for 10 min, extracts were tested for low-molecular-weight (LMW) thiol levels. Prepared extracts (50 μL) were derivatized with SBD-F fluorophore (Sigma-Aldrich, St. Louis, USA). Low-molecular-weight thiols were separated by isocratic HPLC using the method described in Masi et al. (2002). The mobile phase was 3% methanol in 75 mM ${\text{NH}}_{4}^{+}$ formiate, pH 2.9.

### Identification and quantification of glucosinolates

Glucosinolate extraction was performed using a modification of the protocol by Argentieri et al. (2011). To avoid the myrosinase activity in the samples, glucosinolates were extracted from 6 g of plant material boiled for 4 min in 18 mL of a methanol/water solution in the ratio 70:30 (v/v). To this solution, sinigrin (1.26 mg/mL) was added as internal standard. To obtain the complete extraction of glucosinolates, the plant material remained after sample filtration was re-extracted with 7.5 mL of 70% (v/v) methanol for 4 min. The two extracts from each sample were then combined and purified through a SPE (Solid-Phase Extraction) column (0.8 × 4 cm, Agilent Technologies) prepared with 0.256 g of an ion-exchange resin (DEAE-SEPHADEX-A25) re-inflated in 4 mL of a 0.5 M Na-acetate buffer solution (pH = 5). The column was first washed with 1 mL deionized H_2_O, and then loaded with 2.5 mL extract containing the internal standard. The further purification steps included two washings with 2 mL 70% (v/v) methanol, one washing with 2 mL deionized H_2_O and one with 0.5 mL 0.02 M Na-acetate buffer solution (pH = 5). The column was then plugged and treated overnight with the sulfatase enzyme (41.6 mg/mL) from *Heliax pomatia*-Type 1 to convert glucosinolates in the corresponding desulfated derivatives. Subsequently, the desulfated derivatives were eluted from the column by washing it with 2 mL of deionized water twice.

The analysis of glucosinolates was performed in HPLC-MS and the Electrospray Ionization (ESI) as a source in the full scan positive ion-mode. The analysis of the fragmentation patterns of spectra shown in Table 1S was realized through the Turbo Detection Data Scanning (TDDS) function. The chromatographic separation was performed using a column Eclipse XDB C-8 5 μm 2.1 × 150 mm. The mobile phase consisted of mixture of water with 1% (v/v) formic acid (eluent A) and acetonitrile with 0.5% (v/v) acetic acid (eluent B). The flow rate was 200 μL min^−1^ and the volume of each sample injected was 10 μL. For the quantification of glucosinolates, glucoerucin was used as reference standard at different concentration levels.

### Identification and quantification of polyphenols

Polyphenols were extracted from frozen radish tissues using a methanol:water (1:1, v/v) solution in ultrasonic bath for 15 min. The ratio of plant material to mixture was 1:10 (w/v). The extracts were then filtered (0.45 μm, Millipore). The extraction method was validated verifying the recovery percentage of chlorogenic acid and rutin in replicates of leaf and root samples.

Qualitative and quantitative analyses of polyphenols was performed both via HPLC-MS and HPLC-DAD. For the separation of polyphenols, an Eclipse Plus C-18 column (3.5 μm × 2.1 mm × 150 mm, Agilent) was used in HPLC system Varian 212 at 35°C. The column was eluted with a gradient of formic acid (0.1%, v/v, eluent A) and acetonitrile (100%, eluent B), at a flow rate of 200 μL min^−1^. The gradient was as follows: starting with 90% A and 10% B, then in 20 min to 54% B and B and 100% B until 23 min. Re-equilibration time to initial conditions was from 21 to 28 min.

The identification and quantification of the main polyphenol compounds in the samples was achieved via Ion Trap Mass Spectrometry (Varian 500 MS) coupled to the HPLC system, by comparison with appropriate standards (chlorogenic acid for phenols, rutin for flavonoids) and analysis of the fragmentation patterns of spectra (Table 2S) through the TDDS function. ESI was used as source in negative ion-mode, and the mass range considered was 50–3500 uma. The volume of each sample injected was 10 μL.

### Free amino acids

Free amino acids, including Se-amino acids, were extracted from frozen radish leaves and roots (500 mg) using 0.1 M HCl. The ratio of plant material to mixture was 1:4 (w/v). The extracts were centrifuged at 4°C for 10 min at 10,000 rpm. The supernatants were collected and then filtered (0.45 μm, Millipore). Qualitative and quantitative analyses of amino acids were performed via HPLC-MS using a ZORBAX Eclipse Plus AAA column (3.5 μm × 3 × 150 mm). The column was eluted with a gradient of a water solution of formic acid (1%, v/v, eluent A) and acetonitrile (100%, eluent B), at a flow rate of 200 μL min^−1^. The volume of each sample injected was 10 μL. The gradient was as follows: starting with 88% A/12% B for 8.30 min, then in 9 min to 75% B and isocratic until 11 min. Re-equilibration time to initial conditions was from 11.18 to 14.30 min.

The identification and quantification of the amino acids in the samples was achieved via Ion Trap Mass Spectrometry (Varian 500 MS) coupled to the HPLC system, by comparison with appropriate standards and analysis of the fragmentation patterns of spectra (data not shown) through the TDDS function. For the HPLC-MS analysis, ESI in positive ion mode (50-350 uma) was used. For the identification and quantification of the amino acids the reference standards consisted of these amino acids: Alanine, Arginine, Asparagine, Aspartic acid, Cysteine, Glutamine, Glutamic acid, Glycine, Histidine, Isoleucine, Leucine, Lysine, Methionine, Phenylalanine, Proline, Serine, Threonine, Tryptophan, Tyrosine, Valine and Selenomethionine, Selenocystine, Se-Methyl-Selenocysteine.

### Analysis of soluble proteins

Protein levels in radish leaves and roots were determined using the Bradford method 1976. Frozen samples (200 mg) were ground in a mortar with liquid nitrogen and extracted with phosphate buffer (pH 7.8) in the ratio 1:10. Samples were centrifuged for 20 min at 14,000 rpm at 4°C. The supernatant was harvested and 10 μL of extract were used for the protein assay. Data of protein content were obtained by comparing the values measured at λ = 595 with those provided by a reference calibration curve prepared with bovine serum albumin (BSA) at different dilutions.

### Gene expression via qRT-PCR

For quantitative Real-Time PCR experiments, RNA was extracted from three individual samples of roots (white roots) and leaves of radish plants grown in hydroponics under the following experimental conditions: control (0 Se), Se 10 μM, Se 40 μM. RNA extraction was performed using a phenol/chloroform protocol according to Sambrook and Russell (2001). All the cDNAs were prepared from 3 μg of RNAs using 200 U of ImProm-II^™^ Reverse Transcriptase (Promega, Milano, Italy) and oligodT as primers in 20 μl reaction volume. Mixtures were incubated at 37°C for 60 min, 70°C for 5 min, and 4°C for 5 min to stop the RT reaction. Specific primer pairs for sequences were designed on conserved sequences among *Brassicaceae* spp. (Table 3S) and tested for their activity at 58–67°C by conventional PCR. qRT-PCR analyses were performed using a thermal cycler 7300 Real-Time PCR System (Applied Biosystem) equipped with a 96 well plates system with the SYBR green PCR Master Mix reagent (Applied Biosystem). Each qPCR reaction (10 μl final volume) contained 1 μl of diluted cDNA (1:10), 1 μL of primer couple (10 μM), and 5 μl of 2 × SYBR Green PCR Master Mix according to the manufacturer's instructions. The following thermal cycling profile was used for all PCRs: 95°C for 10 min, 50 cycles of 95°C for 15 sec, 60°C for 1 min. The gene expression analysis for each biological replicate was evaluated in two technical replicates (only one set of data is shown in Figures).

All quantifications were normalized to the actin gene used as housekeeping gene and amplified in the same conditions. The obtained CT values were analyzed with the Q-gene software by averaging three independently calculated normalized expression values for each sample. Expression values are given as the mean of the normalized expression values of the triplicates, calculated according to Eq. 2 of the Q-gene software (Muller et al., 2002).

### Determination of total Se, C, N, and S in soil

Samples of soil dried at room temperature were analyzed for carbon (C), nitrogen (N), and sulfur (S) content using an elemental analyzer (Vario MACRO CNS, Hanau, Germany). For total Se determination, soil samples were extracted with HNO_3_/HCl (ratio 1:3 v/v) and warmed until boiling for 30 min under agitation. Samples were then filtered (0.45 μm, Millipore), and the quantification of Se was performed via ICP-AES as described previously (Fassel, 1978).

### Statistical analysis

Analysis of variance (ANOVA) was performed using the SPSS software, and was followed by pair-wise *post-hoc* analyses (Student-Newman-Keuls test) to determine which means differed significantly at *p* < 0.05 (±SD).

## Results

### A. soil experiment

#### Plant growth in response to Se fertilization

Foliar application of selenate to radish plants did not injure plant fresh weight when Se was furnished at 5 or 10 mg per plant, but rather was associated with an increase in the dry weight of leaves and roots (Table 1). In plants treated with 20 mg Se per plant, the fresh weight of leaves, and roots was reduced by about 34 and 11%, respectively, compared to control plants. The dry weight of leaves and roots was also decreased by the supply of 20 mg Se per plant (by 35 and 18%, respectively).

**Table 1:** **Fresh (FW) and dry weight (DW), Se concentration, total Se content (on FW basis), S concentration, sulfate concentration, cysteine (Cys) and glutathione (GSH) content in leaves, and roots of radish plants cultivated in soil**.

	**Leaves**			
	**Se treatment (mg per plant)**			
	**0**	**5**	**10**	**20**
Fresh weight (g)	14.61 ± 1.52a	13.80 ± 1.2a	14.61 ± 2.65a	9.62 ± 0.91b
Dry weight (g)	1.46 ± 0.06b	1.77 ± 0.06a	1.83 ± 0.05a	1.19 ± 0.07c
Se (μg g^−1^ DW)	2.42 ± 1.20d	346.5 ± 48.8c	725.22 ± 170.67b	1299.71 ± 390.94a
Total Se (μg)	0.35 ± 0.11c	78.59 ± 10.1b	166.23 ± 14.25a	191.3 ± 25.30a
S (mg g^−1^ DW)	6.02 ± 1.52c	9.12 ± 0.89bc	10.50 ± 1.93ab	12.20 ± 1.82a
Sulfate (mg g^−1^ DW)	2.35 ± 0.36c	7.66 ± 1.41b	17.08 ± 2.67a	14.49 ± 3.93a
Cys (nmol g^−1^FW)	12.95 ± 0.49a	11.45 ± 2.43a	11.79 ± 0.71a	12.15 ± 2.85a
GSH (nmol g^−1^FW)	137.64 ± 14.43b	155.02 ± 28.86ab	163.23 ± 32.52ab	212.37 ± 51.38a
	**Roots**			
	**Se treatment (mg per plant)**			
	**0**	**5**	**10**	**20**
Fresh weight (g)	25.40 ± 2.6a	27.56 ± 1.65a	29.10 ± 4.32a	22.56 ± 1.08b
Dry weight (g)	1.82 ± 0.17b	2.69 ± 0.30a	2.22 ± 0.25a	1.18 ± 0.14c
Se (μg g^−1^ DW)	0.41 ± 0.03c	26.42 ± 4.32b	90.83 ± 15.01a	111.35 ± 31.02a
Total Se (μg)	0.000 ± 0.000c	6.93 ± 0.90b	15.38 ± 2.50a	6.87 ± 1.86b
S (mg g^−1^ DW)	4.14 ± 0.71a	4.18 ± 0.54a	4.15 ± 0.20a	4.14 ± 0.46a
Sulfate (mg g^−1^ DW)	2.06 ± 0.32a	1.64 ± 0.17a	1.81 ± 0.21a	1.16 ± 0.38b
Cys (nmol g^−1^FW)	2.19 ± 0.44b	5.35 ± 2.18a	7.46 ± 1.08a	2.77 ± 1.24b
GSH (nmol g^−1^FW)	29.27 ± 2.67b	51.76 ± 6.23a	44.66 ± 3.06a	35.10 ± 4.59b

Data represent the mean of 10 biological replicates for FW and DW, and five biological replicates for the remaining determinations. Different letters along rows indicate significant differences (p < 0.05, ±STD) among treatments.

#### Selenium accumulation and effects of Se application on sulfur, sulfate, glutathione, and cysteine levels

The Se concentration in leaves of radish plants supplied with selenate positively correlated with the dosage of Se supplied. In roots, a similar pattern of Se accumulation was observed as in leaves, even though differences in Se concentrations were not significant between plants treated with 10 and 20 mg Se per plant (Table 1). Generally, Se concentration in root tissues was lower than that measured in leaves. The total content of Se in leaves and roots of radish plants, reported on a fresh weight basis, followed a similar trend as Se concentration, with the exception of total Se content values in roots of radishes treated with 20 mg Se per plant, which did not further increase because of root biomass reduction by Se (Table 1). Maximum total contents of Se were detected in leaves of plants sprayed with 10–20 mg Se per plant and in roots of plants treated with 10 mg Se per plant.

Treating plants with increasing selenate dosages led to an increase in sulfur (S) concentration in leaves (2-fold higher in plants treated with 20 mg Se per plant compared to the controls), while no effect on S concentration was detected in roots (Table 1). The Se/S concentration ratio calculated in leaf tissues increased from 0.04 in plants sprayed with 5 mg Se per plant to 0.1 in plants exposed to 20 mg Se per plant. In roots the Se/S concentration ratio in the same plants increased 4-fold Sulfate accumulated at very high levels in leaves of plants treated with selenate, whereas in roots a decrease of sulfate concentration (minus 43%) was evident when plants were treated with the highest selenate dosage (Table 1).

The application of selenate to radish plants did not affect the content of cysteine (Cys) in leaves, but increased it by 2.5–3.5-fold in roots of plants sprayed with either 5 or 10 mg Se per plant (Table 1). Leaf glutathione (GSH) level in plants sprayed with 20 mg Se per plant was significantly higher than in the controls (Table 1). In roots, accumulation of GSH followed the same trend as Cys (Table 1).

#### Effects of Se fertilization on glucosinolate (GLS) content and profile

The main glucosinolates identified in leaves of radish plants were glucoraphanin, glucoraphasatin, glucobrassicin, and neoglucobrassicin. In roots, dimeric-4 mercaptobutyl (DMB-GLS) was additionally identified. The content of total leaf GLSs in plants sprayed with 5 mg Se per plant was comparable to that measured in the controls, but it was 20% higher than in plants exposed to higher selenate dosages (Figure 1A). Glucoraphanin and glucobrassicin accounted for the main leaf GLSs and their increase in content was observed in plants treated with 5 mg Se per plant (10–18% higher compared to the controls) (Figure 1B). Neoglucobrassicin was more accumulated in plants exposed to 20 mg Se per plant (~2-fold higher) compared to the control, whereas glucoraphasatin level was affected by treatment with 10 and 20 mg Se per plant (reduced by 42 and 38%, respectively).

In roots, GLSs accumulated more in plants sprayed with 20 mg Se per plant (55% higher) (Figures 1C,D). The increase was mainly due to the increase in glucoraphanin (3-fold higher than the control).

#### Effects of Se fertilization on nitrogen, nitrate, amino acid, and protein contents

**Figure 2:**
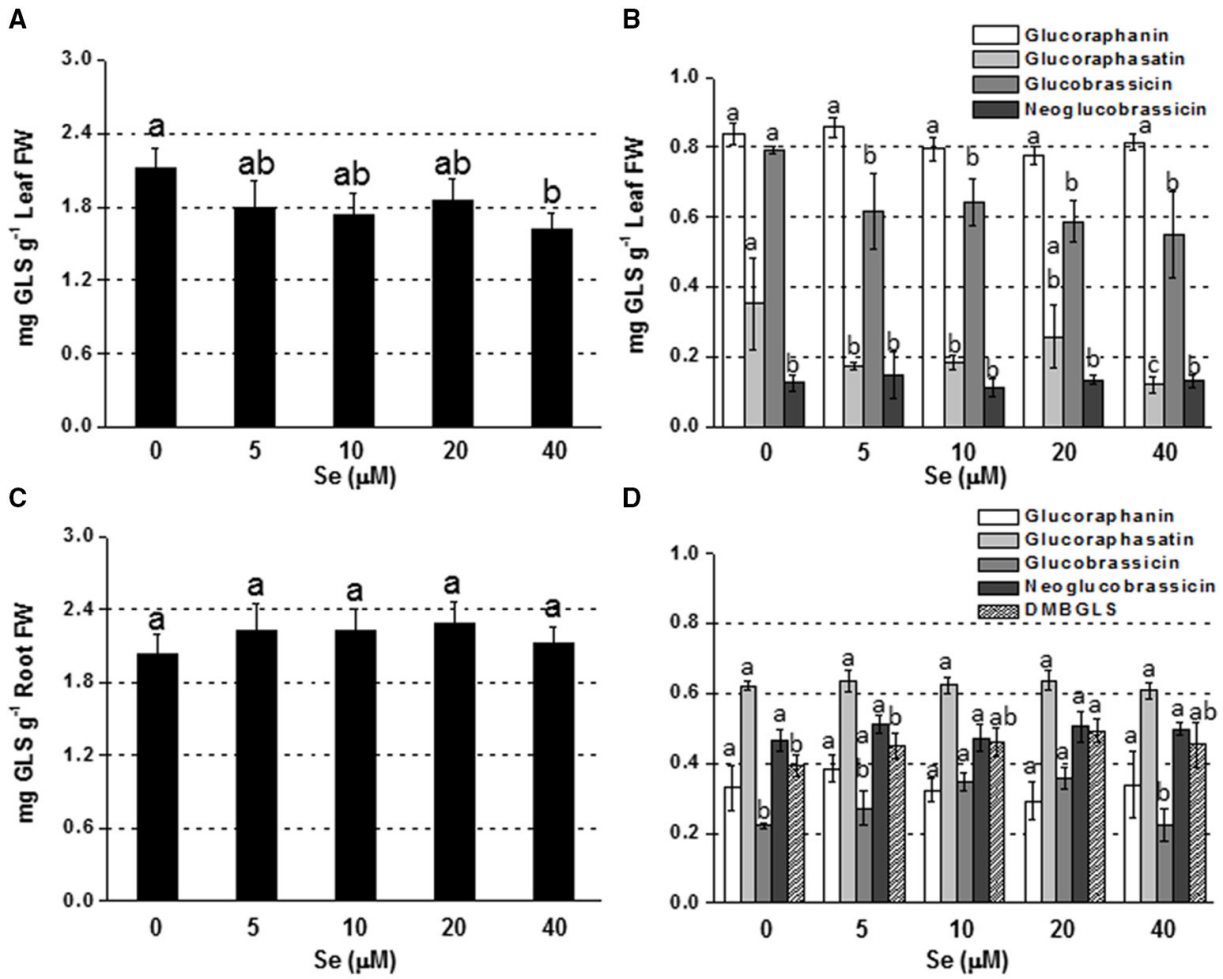
**Concentration of total (A,C) and individual (B,D) glucosinolates (GLSs) in radish leaves (A,B) and roots (C,D) grown in hydroponics supplied with selenate concentrations ranging from 0 to 40 μM Se**. Letters above bars indicate significant differences between the means (*n* = 3, ±*SD*, *p* < 0.05). In **(B,D)** the statistical analysis was performed between values of GLS concentration vs. Se concentration for each individual GLS.

Foliar fertilization of radish plants with either 5 and 10 mg Se per plant significantly reduced the leaf content of total N and nitrate (${\text{NO}}_{3}^{-}$) (Figure 2SA). On the contrary, Se application promoted total N and ${\text{NO}}_{3}^{-}$ accumulation in roots of plants treated with 10 or 20 mg Se per plant, or 20 mg Se per plant in the case of ${\text{NO}}_{3}^{-}$, respectively (Figure 2SB). The leaf protein content was reduced by high Se treatment, while it was enhanced in roots by 10 and 20 mg Se per plant (Figures 2SC,D).

When radish plants were sprayed with 10 or 20 mg Se per plant they showed enhanced total amino acids content in foliar tissues, whereas no appreciable variation was observed in roots (Table 2).

**Table 2:** **Effects of selenate treatment on the content of selected amino acids in leaves and roots of radish plants cultivated in soil**.

**Amino acid (% w/w)**	**Leaves**			
	**Se treatment (mg per plant)**			
	**0**	**5**	**10**	**20**
Phenylalanine	3.84 ± 1.56b	5.45 ± 0.93b	17.04 ± 5.91a	14.36 ± 3.08a
Isoleucine	0.83 ± 0.01c	0.78 ± 0.10c	1.32 ± 0.22b	2.38 ± 0.62a
Leucine	0.28 ± 0.01b	0.20 ± 0.14b	0.76 ± 0.36a	0.75 ± 0.26a
Histidine	9.06 ± 0.81b	11.98 ± 2.36ab	8.26 ± 0.78b	11.18 ± 0.76a
Tyrosine	0.14 ± 0.03b	0.08 ± 0.01b	0.37 ± 0.17a	0.33 ± 0.15a
Tryptophan	0.39 ± 0.06c	0.27 ± 0.10c	0.83 ± 0.31b	1.40 ± 0.43a
Asparagine	3.67 ± 1.08a	4.63 ± 1.25a	4.34 ± 1.19a	3.29 ± 0.24a
Glutamine	33.43 ± 17.33b	26.15 ± 5.85b	80.74 ± 24.57a	102.30 ± 24.73a
Valine	9.55 ± 1.34a	7.54 ± 0.73a	10.01 ± 2.71a	8.47 ± 2.11a
Proline	40.85 ± 7.36a	27.86 ± 1.91b	22.57 ± 8.21b	16.50 ± 5.27b
Lysine	1.21 ± 0.36a	1.22 ± 0.11a	1.07 ± 0.28a	0.96 ± 0.16a
Se-methylselenocysteine	−	5.83 ± 1.11b	7.23 ± 1.98b	9.30 ± 1.94a
Se-cysteine	−	−	−	−
Se-methionine	−	−	−	−
Total	2.03 ± 0.15b	2.05 ± 0.13b	2.47 ± 0.23a	2.49 ± 0.27a
**Amino acid (% w/w)**	**Roots**			
	**Se treatment (mg per plant)**			
	**0**	**5**	**10**	**20**
Phenylalanine	9.73 ± 3.32b	9.10 ± 1.02b	10.76 ± 2.01ab	14.93 ± 2.56a
Isoleucine	17.21 ± 2.78b	12.83 ± 2.62b	13.88 ± 3.09b	23.45 ± 3.19a
Leucine	3.67 ± 0.94b	3.51 ± 0.08b	3.73 ± 0.45b	6.19 ± 0.02b
Histidine	7.13 ± 0.79a	8.75 ± 0.75a	7.75 ± 1.32a	6.72 ± 1.36a
Tyrosine	1.49 ± 0.34b	1.23 ± 0.25b	1.40 ± 0.20b	2.17 ± 0.43a
Tryptophan	1.66 ± 0.40ab	1.82 ± 0.11ab	1.43 ± 0.23b	2.57 ± 0.54a
Asparagine	4.26 ± 0.39a	3.61 ± 0.89a	4.19 ± 1.10a	4.52 ± 1.33a
Glutamine	97.09 ± 3.69b	75.07 ± 1.12c	78.70 ± 7.02c	121.45 ± 2.98a
Valine	50.06 ± 3.36	48.10 ± 10.82	41.42 ± 11.70	47.71 ± 16.98
Proline	98.74 ± 24.96ab	101.32 ± 13.33a	65.95 ± 22.31b	73.83 ± 13.98b
Lysine	0.72 ± 0.13a	0.58 ± 0.15a	0.68 ± 0.25a	0.69 ± 0.11a
Methionine	2.08 ± 0.14a	1.49 ± 0.11b	1.66 ± 0.10b	1.77 ± 0.29ab
Se-methylselenocisteine	−	1.73 ± 1.09b	1.62 ± 0.11b	3.34 ± 1.05a
Se-cysteine	−	−	−	−
Se-methionine	−	−	−	−
Total	3.62 ± 0.16a	3.56 ± 0.20a	3.13 ± 0.33a	3.80 ± 0.27a

Data are expressed as mg amino acid per 100 mg tissue fresh weight (FW). Different letters along rows indicate significant differences (n = 4, ±STD, p < 0.05) among treatments.

The leaf concentration of most amino acids increased after selenate treatment, with the exception of asparagine, valine, lysine and proline (Table 2). The level of proline in particular, was dramatically reduced by all Se dosages, while the amount of glutamine was strongly increased by 10 and 20 mg Se per plant. In roots, the content of several individual amino acids increased after plant exposure to high Se. Interestingly, the seleno-amino acid Se-methylselenocysteine (MSeC) was identified and determined in both roots and leaves of radish plants treated with selenate, while seleno methionine (SeMet) and seleno cysteine (SeCys) were not detected. The leaf and root concentration of MSeC was similar between plants treated with 5 and 10 mg Se per plant, and increased only in plants supplied with 20 mg Se per plant.

#### Effects of Se fertilization on polyphenol content and profile

The identification of phenolic compounds was based on mass spectra obtained under electron spray ionization (ESI) conditions in negative ion mode and was confirmed by the comparison of relative fragmentations with the current literature. In radish, the main flavonol identified was kaempferol, conjugated to glucose or rhamnose, while the most representative hydroxycinnamic acids were coumaric, malic, sinapic, and ferulic acids, sometimes found in conjugation with other hydroxycinnamic acids. Polyphenol concentration was calculated using the calibration curves of rutin for flavonoids and chlorogenic acid for hydroxycinnamic acids.

Foliar Se application to plants differentially influenced the accumulation of individual phenolic compounds in leaves and roots. In leaves, 11 main polyphenols were identified (Table 3). Five of them were flavonols and kaempferol-derivatives, the remaining six were hydroxycinnamic acids. With the exception of caffeic acid, kaempherol-3-O-arabinoside-7-O-rhamnoside, kaempherol-3-ramnosil glucoside, kaempherol-3,7-diramnoside, sinapi\c acid, which were found to increase at specific Se dosages, the other identified phenolics did not show a variation in content (kempherol-7-O-rhamnoside and cumaric acid), or otherwise a decrease (kaempherol-3-glucoside, ferulic acid, feruilmalate sinapoilmalate). The maximum leaf content in polyphenols was observed in plants sprayed with 5 mg Se per plant (10% higher than the controls).

**Table 3:** **Content of phenolic compounds identified in leaves and roots of radish plants cultivated in soil**.

**Polyphenol *(μ*g kg^−1^ FW)**	**Leaves**			
	**Se treatment (mg per plant)**			
	**0**	**5**	**10**	**20**
Kaempherol-3-glucoside	25 ± 4a	19 ± 5a	9 ± 4b	9 ± 3a
Kaempherol-7-O-rhamnoside	7 ± 3a	8 ± 3a	7 ± 2a	8 ± 3a
Caffeic acid	14 ± 3b	30 ± 5a	29 ± 5a	2 ± 1c
Kaempherol-3-rhamnosil glucoside	58 ± 13b	57 ± 10b	32 ± 16b	80 ± 24a
Kaempherol-3-O-arabinoside-7-O-rhamnoside	24 ± 3b	34 ± 4b	28 ± 3b	51 ± 10a
Kaempherol-3,7-dirhamnoside	57 ± 12b	92 ± 16a	88 ± 15a	90 ± 23a
Coumaric acid	134 ± 20a	111 ± 25a	160 ± 19a	132 ± 15a
Sinapic acid	6 ± 1b	11 ± 2a	4 ± 2b	4 ± 1b
Ferulic acid	151 ± 5a	170 ± 8a	126 ± 13b	56 ± 10b
Feruilmalate	60 ± 13a	60 ± 11a	59 ± 15a	30 ± 18b
Sinapoilmalate	16 ± 3a	14 ± 4a	14 ± 3a	7 ± 1b
Total	552 ± 13a	606 ± 15b	556 ± 10a	469 ± 15c
**Polyphenol *(μ*g kg^−1^ FW)**	**Roots**			
	**Se treatment (mg per plant)**			
	**0**	**5**	**10**	**20**
Kaempherol-3-rhamnosil glucoside	20 ± 3a	24 ± 3a	17 ± 4a	24 ± 3a
Coumaric acid	56 ± 12a	21 ± 10b	9 ± 5b	10 ± 3b
Ferulic acid	53 ± 14a	30 ± 8ab	19 ± 8b	25 ± 12b
Kaempherol-7-O-rhamnoside	2 ± 1a	2 ± 0a	2 ± 0a	2 ± 1a
Total	131 ± 10a	77 ± 7b	47 ± 6c	61 ± 6bc

Different letters along rows indicate significant differences (n = 4, ±STD p < 0.05) among treatments.

In roots, only four main phenolic compounds were detected: kampferol-7-O-rhamnoside, coumaric acid, ferulic acid, kaempferol rhamnosil glucoside (Table 3). While no change in content due to Se treatment was evident for the flavonols kampferol-7-O-rhamnoside and kaempferol rhamnosil glucoside, the level of coumaric and ferulic acids decreased in plants exposed to selenate. As a result, the amount of total phenols in roots was reduced by about 40–60% in Se-treated plants.

#### Selenium, nitrogen, carbon, and sulfur content in soil after foliar Se fertilization

Selenium concentration was very low (< 0.5 mg kg^−1^) in soil collected from pots where control plants were cultivated (Table 4S). In soil sampled from pots used to grow Se-treated plants, a non-significant increase of Se concentration was observed, and values remained low (< 3 mg kg^−1^). N percentage in soil did not change significantly, while S percentage decreased in soil where plants sprayed with 20 mg Se per plants were grown. With respect to C content, no appreciable change was observed, except for a 35% increase in soil used for cultivation of plants treated with 10 mg Se per plant in relation to 5 mg Se/ plant.

### B. hydroponic experiment

#### Effects of Se on plant growth parameters

The supply of either 10 or 20 μM Se in the nutrient solution enhanced the fresh biomass of radish leaves (25% higher) and red roots (~20% higher) (Table 4). However, 40 μM Se caused a reduction of plant growth, and the effect was more pronounced for the red roots (minus 50%). Under hydroponic conditions, plants developed also white roots, whose biomass production in fresh weight was stimulated by all Se doses (~2-fold higher than the controls). No appreciable changes in dry weight were measured in leaves of Se-treated plants compared to the control, while root growth was affected for both red roots (35% lower by 10 μM Se,) and white roots (35% lower by 10 and 20 μ M Se, Table 4).

**Table 4:** **Fresh (FW) and dry weight (DW), Se concentration, total Se content (on a FW basis), S concentration, sulfate concentration, cysteine (Cys), and glutathione (GSH) content in leaves and roots of radish plants cultivated in hydroponics**.

	**Leaves**				
	**Se treatment (μM)**				
	**0**	**5**	**10**	**20**	**40**
Fresh weight (g)	13.40 ± 0.88b	13.86 ± 0.97b	16.71 ± 1.33a	16.82 ± 1.31a	11.16 ± 1.29c
Dry weight (g)	1.57 ± 0.27ab	1.58 ± 0.18a	1.88 ± 0.19a	1.35 ± 0.12b	1.41 ± 0.14b
Se (μg g^−1^ DW)	n.d.	14.42 ± 13.19c	44.10 ± 13.19c	88.55 ± 16.73b	241.89 ± 34.58a
Total Se (μg)	n.d.	2.59 ± 0.64c	9.32 ± 1.65b	9.59 ± 2.75b	43.09 ± 7.89a
S (mg g^−1^ DW)	6.16 ± 0.50c	7.19 ± 0.66b	8.38 ± 0.62ab	8.68 ± 0.60a	9.50 ± 0.85a
Sulfate (mg g^−1^ DW)	2.84 ± 0.41c	5.52 ± 2.30b	9.46 ± 0.07a	4.68 ± 0.82b	6.17 ± 1.34b
Cys (nmol g^−1^FW)	11.58 ± 3.47a	7.02 ± 1.18a	4.66 ± 0.75b	4.82 ± 1.15b	4.98 ± 1.83b
GSH (nmol g^−1^FW)	136.72 ± 29.12a	91.82 ± 2.23b	83.95 ± 8.75bc	87.84 ± 21.93bc	67.59 ± 8.07c
	**Roots**				
	**Se treatment (μM)**				
	**0**	**5**	**10**	**20**	**40**
Red root fresh weight (g)	13.66 ± 0.77b	12.05 ± 0.98b	16.48 ± 1.57a	15.97 ± 1.08a	6.84 ± 1.04c
White root fresh weight (g)	0.81 ± 0.12b	1.56 ± 0.24a	1.20 ± 0.19a	1.53 ± 0.25a	1.31 ± 0.15a
Red root dry weight (g)	1.16 ± 0.19a	1.19 ± 0.13a	1.26 ± 0.078a	1.10 ± 0.07a	0.73 ± 0.12b
White root dry weight (g)	0.103 ± 0.008a	0.110 ± 0.006a	0.122 ± 0.017a	0.070 ± 0.007b	0.067 ± 0.014b
Red root Se (μg g^−1^ DW)	n.d.	6.60 ± 0.14c	23.22 ± 12.96b	34.65 ± 14.83b	84.32 ± 30.37a
White root Se (μg g^−1^ DW)	n.d.	10.03 ± 4.71d	20.67 ± 2.90c	49.79 ± 13.71b	86.76 ± 17.56a
Total red root Se (μg)	n.d.	0.77 ± 0.22c	2.23 ± 0.32b	2.62 ± 0.26b	6.56 ± 2.45a
Total white root Se (μg)	0.00 ± 0.00c	0.007 ± 0.001b	0.25 ± 0.10a	0.16 ± 0.05a	0.29 ± 0.07a
Red root S (mg g^−1^ DW)	3.62 ± 0.25b	4.07 ± 0.43ab	4.89 ± 0.46a	4.26 ± 0.47ab	4.31 ± 0.73ab
White root S (mg g^−1^ DW)	5.08 ± 0.31b	4.19 ± 0.73ab	4.28 ± 0.56a	4.66 ± 0.50ab	4.09 ± 0.51ab
Sulfate (mg g^−1^ DW)	0.59 ± 0.13ab	0.72 ± 0.21ab	0.77 ± 0.14a	0.48 ± 0.05b	0.57 ± 0.11ab
Cys (nmol g^−1^FW)	9.79 ± 2.17b	12.42 ± 2.79ab	16.84 ± 2.19a	12.50 ± 4.90ab	13.82 ± 3.02ab
GSH (nmol g^−1^FW)	136.97 ± 26.12a	126.57 ± 6.23ab	129.08 ± 30.36ab	98.45 ± 29.16ab	98.45 ± 6.62b

Data represent the mean of 20 biological replicates for Fresh and Dry weight, and five biological replicates for the remaining determinations. Different letters along rows indicate significant differences (p < 0.05, ±STD) among treatments. For roots, sulfate, Cys, and GSH were measured only in the red root. n.d., not determined.

#### Selenium, sulfur, sulfate, glutathione, and cysteine accumulation in relation to Se fertilization rate

Radish plants had higher Se concentrations in leaves than in red or white roots, among which no differences were evident (Table 4). The concentration ratio [Se]_leaf_/[Se]_root_ ranged from 2.2 in plants supplied with 5 μM Se to 2.9 in plants treated with 40 μM Se, in the case of red roots, or from 1.4 to 2.8 for white roots. The total content of Se in leaves and red roots of radish plants, reported on a fresh weight basis, followed a similar trend as Se concentration in tissues, while in white roots it was not significantly different among plant treated with 10–40 μM Se (Table 4). The plants grown in the presence of 40 μM Se exhibited the highest values of Se content in leaves (43 μg), as well as in roots (about 6.6 μg in red roots).

Sulfur concentration in leaves and red roots tended to increase with increasing Se dosages in the nutrient solution. In white roots, no significant variation in S concentration was observed. Values of Se/S ratio were similar among plant organs. At 40 μM Se, the Se/S ratio ranged between 0.02 and 0.025, vs. the Se/S ratio applied of 0.08.

The leaf concentration of sulfate was higher in Se-treated plants than in the controls (65–117% higher), with maximum values measured at the 10 μM Se treatment. In red roots, no significant changes in sulfate concentration happened; it is worth noting that the highest concentration measured was for the 10 μM Se treatment, as in leaves.

The analysis of thiol content revealed a dramatic reduction in Cys and GSH levels in leaves of Se-treated radish plants. In the presence of 40 μM Se, this reduction was about 50% for both Cys and GSH. In red roots of plants treated with the same Se concentration, the GSH amount decreased as well, by about 30%. On the contrary, Cys content in roots was generally unaffected by Se, except for an increase observed in red roots treated with 10 μM Se.

#### Effects of Se fertilization on glucosinolate (GLS) content and profile

Adding Se at high concentration (40 μM) to the nutrient solution resulted in a drop in leaf GLS production, whereas no change was reported at lower Se doses (Figure 2A). The main individual GLSs affected by Se treatment were glucoraphasatin (65% lower) and glucobrassicin (30% lower) (Figure 2B).

In roots, a general trend of increasing GLS accumulation was found in response to Se exposure, although values were not significantly different from the control (Figure 2C). The level of glucobrassicin, in particular, was higher in plants treated with 10 or 20 μM Se, while dimeric-4 mercaptobutyl (DMB-GLS) concentration was boosted by 20 μM Se compared to the control (Figure 2D).

#### Effects of Se fertilization on amino acid and phenolic content and profile

The total content of amino acids increased in leaves of plants treated with Se concentrations as high as 20 or 40 μM, while an opposite trend was observed in roots (Table 5). Differences in proline levels accounted for the main part of these differences. As reported for radish plants grown in soil, MSeC was the only Se-amino acid identified in plants cultivated in hydroponics under Se treatment, but its level in leaves did not vary in response to different Se dosages. In roots, values of MSeC were similar among plants grown in the presence of 10–40 μM Se, and up to 2-fold higher than those measured in plants treated with a low Se level (5 μM).

**Table 5:** **Effects of selenate treatment on the content of selected amino acids in leaves and roots of radish plants cultivated in hydroponics**.

**Amino acid (% w/w)**	**Leaves**				
	**Se treatment (μM)**				
	**0**	**5**	**10**	**20**	**40**
Phenylalanine	3.36 ± 0.48a	1.79 ± 0.17c	2.71 ± 0.48b	2.21 ± 0.32bc	1.68 ± 0.24c
Isoleucine	0.49 ± 0.11a	0.71 ± 0.14a	0.58 ± 0.05a	0.62 ± 0.15a	0.12 ± 0.02b
Leucine	0.50 ± 0.07a	0.46 ± 0.03a	0.50 ± 0.01a	0.38 ± 0.02b	0.28 ± 0.04b
Histidine	2.17 ± 0.11b	2.63 ± 0.15a	2.60 ± 0.06a	2.88 ± 0.15a	2.84 ± 0.08a
Tyrosine	0.65 ± 0.08a	0.35 ± 0.05b	0.47 ± 0.07b	0.49 ± 0.06b	0.32 ± 0.03c
Tryptophan	1.24 ± 0.16a	0.72 ± 0.12b	0.58 ± 0.21b	0.58 ± 0.43b	0.51 ± 0.10b
Asparagine	1.38 ± 0.15a	1.45 ± 1.25a	1.54 ± 0.17a	1.47 ± 0.08a	1.66 ± 0.23a
Glutamine	25.44 ± 1.34a	22.36 ± 1.53ab	21.31 ± 0.74b	23.91 ± 1.85ab	26.98 ± 2.64a
Valine	3.19 ± 0.09a	2.14 ± 0.25b	2.55 ± 0.44b	2.21 ± 0.11b	1.69 ± 0.40b
Proline	12.15 ± 0.96c	13.32 ± 2.43c	19.70 ± 3.14b	23.99 ± 4.43ab	27.05 ± 1.81a
Methionine	0.25 ± 0.02a	0.19 ± 0.01b	0.25 ± 0.05a	0.19 ± 0.03ab	0.16 ± 0.05b
Se-methylselenocysteine	−	1.15 ± 0.49a	1.63 ± 0.48a	1.24 ± 0.66a	1.52 ± 0.21a
Se-cysteine	−	−	−	−	−
Se-methionine	−	−	−	−	−
Total	50.82 ± 2.16b	47.27 ± 2.03b	54.42 ± 2.00b	60.17 ± 3.17a	64.81 ± 3.14a
**Amino acid (% w/w)**	**Roots**				
	**Se treatment (μM)**				
	**0**	**5**	**10**	**20**	**40**
Phenylalanine	5.58 ± 0.56a	5.69 ± 0.16a	6.05 ± 0.70a	4.74 ± 0.22b	5.42 ± 0.65ab
Isoleucine	5.95 ± 1.66ab	7.34 ± 0.78a	7.25 ± 1.03a	4.33 ± 0.65b	4.16 ± 1.26b
Leucine	1.44 ± 0.26b	2.20 ± 0.10a	2.24 ± 0.45ab	1.57 ± 0.16b	2.07 ± 0.46ab
Histidine	1.83 ± 0.44b	2.72 ± 0.36a	2.48 ± 0.32a	2.43 ± 0.31a	2.54 ± 0.50a
Tyrosine	1.05 ± 0.15a	1.26 ± 0.16a	1.16 ± 0.21a	0.91 ± 0.24a	1.03 ± 0.25a
Tryptophan	2.33 ± 0.47ab	2.67 ± 0.09a	2.58 ± 0.35ab	2.03 ± 0.32ab	1.71 ± 0.42b
Asparagine	1.65 ± 0.48a	1.51 ± 0.22a	1.31 ± 0.15a	1.48 ± 0.14a	1.89 ± 0.35a
Glutamine	21.10 ± 2.07a	20.14 ± 1.64a	25.30 ± 4.72a	21.28 ± 3.09ab	22.66 ± 2.28a
Valine	11.40 ± 1.23ab	14.41 ± 1.79a	13.76 ± 2.25a	8.61 ± 1.30b	8.40 ± 1.78b
Proline	50.06 ± 3.42c	43.45 ± 2.10c	43.03 ± 1.40b	39.44 ± 4.14ab	24.19 ± 3.65a
Methionine	0.86 ± 0.17b	1.31 ± 0.14a	1.25 ± 0.17a	0.79 ± 0.11b	1.12 ± 0.22b
Se-methylselenocysteine	−	0.75 ± 0.06b	1.51 ± 0.33a	1.05 ± 0.12a	1.27 ± 0.35a
Se-cysteine	−	−	−	−	−
Se-methionine	−	−	−	−	−
Total	103.2 ± 2.9a	102.4 ± 2.2a	100.9 ± 3.1a	88.6 ± 3.6b	76.5 ± 2.0c

Data are expressed as mg amino acid per 100 mg tissue fresh weight (FW). Different letters along rows indicate significant differences (n = 5, ±STD, p < 0.05) among treatments.

The leaf concentration of all amino acids that function as precursors of GLSs (phenlylalanine, tyrosine, tryptophan, and methionine) was reduced following plant exposure to Se, even when Se was supplied at 5 μM. This effect was not clearly evident in roots for these aminoacids, as in the presence of specific Se concentrations (5 and 10 μM) Met showed an increase in amount.

With respect to the content of phenolic compounds, Se did not affect these significantly (Table 5S).

#### Differential expression of S- and GLS-related genes in response to Se fertilization

The analysis of transcript accumulation in plants exposed to Se was performed in leaves and white roots, as the red roots were hardly suitable for all the protocol steps.

**Figure 3:**
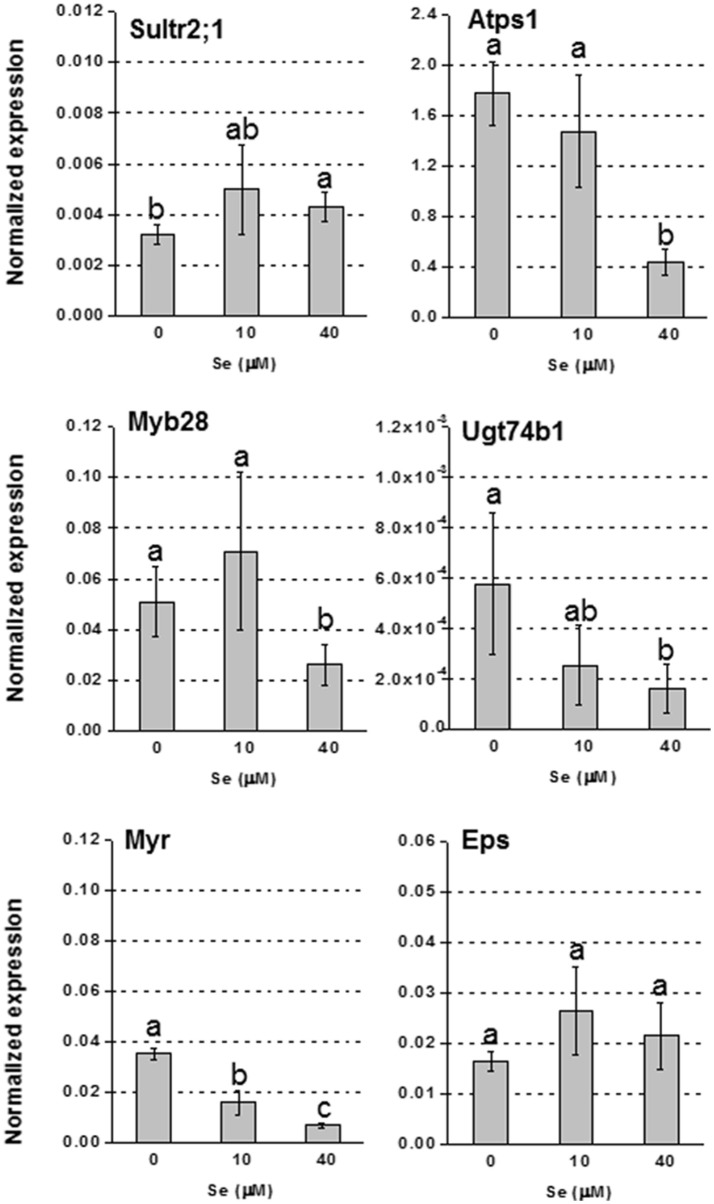
**Expression profiling by real-time RT-PCR of Sultr2;1, Atps1, Myb28, Ugt74B1, Myr, and Eps in leaves of radish plants grown in hydroponics and supplied with selenate concentrations ranging from 0 to 40 μM Se**. Letters above bars indicate significant differences between the means (*n* = 3, ±*SD*, *p* < 0.05).

In foliar tissues, with the exception of the low affinity sulfate transporter gene Sultr2;1 that was up-regulated in plants supplied with 40 μM Se (Figure 3), and the gene Atps4 encoding the ATPS4 isoform of ATP sulfurylase, which was not affected by Se (data not shown), the transcript level of genes involved in S assimilation (Atps1), or GLS biosynthesis (transcription factor Myb28 and glucosyltransferase Ugt74b1) was repressed under high Se (40 μM) (Figure 3). The mRNA abundance of the Myr gene coding for the myrosinase enzyme mediating GLS breakdown was reduced by treatment with 10 μM Se (2-fold lower) and 40 μM Se (4-fold lower), while the expression of the gene coding for the epithio specifier EPS protein, implied in the synthesis of epithionitriles during the hydrolysis of alkenyl glucosinolates, was not significantly altered (Figure 3).

**Figure 4:**
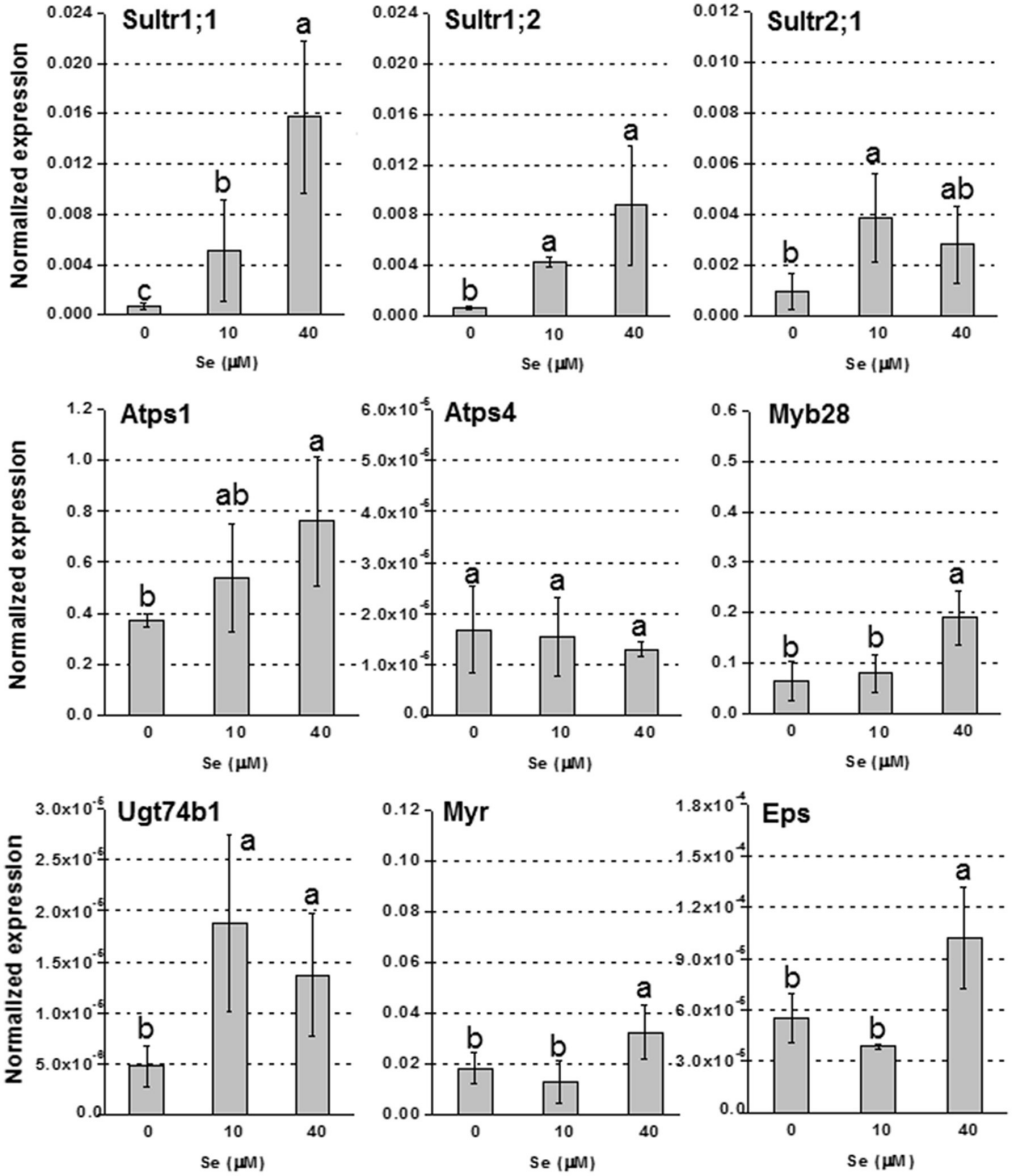
**Expression profiling by real-time RT-PCR of Sultr1;1, Sultr1;2, and Sultr2;1, Atps1, Atps4, Myb28, Ugt74b1, Myr, and Eps in roots of radish plants grown in hydroponics and supplied with selenate concentrations ranging from 0 to 40 μM Se**. Letters above bars indicate significant differences between the means (*n* = 3, ±*SD*, *p* < 0.05).

In roots, the up-regulation of high affinity sulfate transporter genes Sultr1;1 and Sultr1;2, was evident in plants treated with Se, and the effect was more pronounced under 40 μM Se (Figure 4). The transcript level of Sultr2;1 was also stimulated under Se treatment. The genes encoding Atps1, Myb28, Ugt74b1, Myr and Eps, were all more expressed in plants exposed to Se, most of them especially when it was furnished at 40 μM; Atps4, was not affected by Se.

## Discussion

Two methods of Se fertilization were tested to evaluate their efficiency in stimulating accumulation of total Se and organic selenocompounds in radish roots. Furthermore, as Se biofortification should be achieved without compromising the synthesis of other health beneficial molecules (but ideally, while stimulating their synthesis), an additional aim was to evaluate how the two ways of Se supplementation to plants affected plant S, N, and nutraceutical secondary metabolites.

Most of research on Se biofortification has been performed in broccoli, lentils, tomato, and wheat so far (Thavarajah et al., 2008, 2015; Schiavon et al., 2013; Avila et al., 2014; Galinha et al., 2015; Bachiega et al., 2016; Tian et al., 2016). Like broccoli, radish is a member of the *Brassicaceae* family, and contains a wide spectrum of beneficial health compounds (Minich and Bland, 2007). In the case of radish, Se accumulation in the red root, which is the most common edible organ, is a challenge when plants are grown in soil and Se is applied as a foliar spray. Alternatively, Se fertilizers can be applied to soil, but they have the disadvantage to be effective only when soil conditions are uniform. They also need to be reapplied annually and farmers must be carefully instructed on dosages and the method of application (Wu et al., 2015). Another option is radish cultivation in hydroponics, which offers the advantage that roots are directly in contact with Se. However, the amount of water required for plant growth may be limiting in some countries.

In this study, both foliar Se fertilization and hydroponics systems stimulated plant biomass production at low Se dosages. This beneficial effect was observed in previous studies in other plant species (see review by Pilon-Smits et al., 2009). In terms of dry weight, which is the best parameter to evaluate plant productivity, the root of radish developed more in plants sprayed with Se (48% higher at 5 mg Se per plant compared to the controls) than in plants cultivated in hydroponics (Tables 1, 4).

Interestingly, when plants received Se as a spray, Se was also significantly accumulated in roots (Table 1). Se could be mobile in the phloem in both inorganic and organic forms. The transport of Se as organic metabolites is a plausible hypothesis, considering that leaves were directly in contact with Se and chloroplasts are the main intracellular compartments where Se is incorporated into organic compounds via the S assimilation pathway (Sors et al., 2005).

Total Se accumulated in radish roots was low enough to be considered safe for consumption. The intake of about 10 radishes (roughly 280 g of roots) from radish plants cultivated in soil and sprayed with 5 mg Se per plant, or 10 radishes (about 70 g of roots) derived from plants grown in hydroponics and supplied with 40 μM Se, would be sufficient to fulfill the RDA of 70 μg Se stated by both European Food Safety Authority (2014) and USDA (2012). However, a careful consumption of the leafy part of radishes should be performed, even though it is used as forage, as it might result in selenosis symptoms if ingested over long periods. Indeed, Se concentration was very high in leaves, especially when plants were sprayed with Se or furnished in hydroponics with 40 μM Se.

Among beneficial Se-amino acids, MSeC was the only identified in radish plants grown either in soil or hydroponics (Tables 2, 5). This compound has recognized important anticarcinogenic properties (Medina et al., 2001), thus its accumulation in radish roots is a valuable result. Plants sprayed with Se produced more MSeC compared to plants grown in hydroponics, probably because of higher levels of Se in tissues.

Se fertilization also improved S accumulation, especially in leaves, regardless of the Se fertilization method applied (Tables 1, 4). Stimulation of S uptake by low Se dosages was previously observed in other studies (White et al., 2004; Barickman et al., 2013; Schiavon et al., 2015). In radish plants grown in hydroponics, this effect was mainly due to the up-regulation of high affinity sulfate transporter genes, Sultr1;1 and Sultr1;2, while the enhancement of Sultr2;1 transcript abundance may explain the elevated values of S and Se accumulation in foliar tissues of plants grown in hydroponics, as well as the significant [Se]_leaf_/[Se]_root_ ratio. Sultr2;1 plays a role in xylem loading, and thus in root-to-shoot transport of sulfate/selenate (Kawashima et al., 2011; Maruyama-Nakashita et al., 2015). The high leaf-to-root Se partition ratio indicates great Se translocation capacity in radish plants, which may be exploited in other fields beyond biofortification, for instance in phytoremediation technologies.

With respect to S metabolism, the synthesis of some S-metabolites was altered in plants exposed to Se, either when the element was supplied as a foliar spray or added to the nutrient solution (Tables 1, 4). In the case of plants grown in hydroponics, the reduction of Cys, and GSH concentration with the concomitant accumulation of sulfate were observed in leaves. Likely, Se hindered the S assimilation pathway, limiting the reduction of sulfate for the biosynthesis of Cys and then GSH. Indeed, sulfate in Se-treated plants was more accumulated as the inorganic anion sulfate, suggesting that was a relatively lower entry of the sulfate pool into the S assimilation pathway. In roots, the same pattern was evident for GSH at high Se dosages, while Cys was not affected. It is possible that the lower Se concentration in roots did not significantly affect the production of S amino acids (or Cys is more used to form methionine, see Table 1). Unlike plants grown in hydroponics, radish cultivated in soil, and sprayed with Se showed higher production of both Cys and GSH compared to the untreated controls, especially in roots. GSH is a valuable stress marker that has a pivotal role in antioxidant defense systems in plants, by functioning as a precursor of phytochelatins and a free radical scavenger (Grill et al., 1989; Tausz et al., 2004). Its increase in Se-treated plants could be useful to destroy reactive oxygen species (ROS) generated by high levels of inorganic Se in tissues (Ribeiro et al., 2011).

The two methods of Se biofortification caused also different effects on GLS production (Figures 1, 2). GLSs are S-containing glycosides produced by the plant in response to attacks by insects and herbivores, while in humans they exhibit anticarcinogenic properties. However, their content in plants should not exceed the threshold that can become toxic for plants and organisms that feed on them.

In previous studies on Se-GLS interactions, performed mainly in broccoli, either a Se-related reduction in GLSs (Robbins et al., 2005; Barickman et al., 2013) or no variations in their content (Sepúlveda et al., 2013; Tian et al., 2016) were reported. In the same plant species treated with SeO_2_, however, an increase of several GLSs was observed by Thiruvengadam and Chung (2015).

In our study, in plants sprayed with Se no variation in GLS content was observed in leaves, while an increase in all individual GLSs was observed in roots, including the powerful anticarcinogen glucoraphanin (Figures 1B,D). The increase was likely associated with the increase in content of most GLSs' amino acid precursors.

In plants cultivated in hydroponics, Se did not affect GLS level in roots, but decreased it in leaves (Figure 2B). The analysis of GLS-related genes helped to unravel the mechanisms that may explain the pattern of GLS accumulation in these plants. In roots, the transcription factor Myp28 that functions as a major regulator of aliphatic GLS biosynthesis (Augustine et al., 2013), as well as the genes whose promoters it controls (Atps1 and Ugt74b1) (Yatusevich et al., 2010), were up-regulated in roots by Se (Figure 4). The gene Atps1 encodes ATP sulfurylase 1, the major isoform of this enzyme, and implied to be a rate-limiting step for Se assimilation (Pilon-Smits et al., 1999). The up-regulation of this gene was not due to a decrease in S content, but probably to the need of plants to convert inorganic Se accumulating in tissues into less toxic organic forms. The gene Ugt74b1 encodes a glucosyltransferase protein involved in the last step of GLS synthesis (Grubb et al., 2004). The up-regulation of Myb28 leads to stimulation of GLS production (Augustine et al., 2013) likely via up-regulation of genes implied in the process, like Ugt74b1. However, in roots no significant increase of GLS accumulation was observed (Figure 2D). This could be due to two possible factors: first, the roots analyzed for gene expression were the white ones, while the GLS analysis was performed in the edible red roots; second, the Se-induced transcript stimulation of Myr and Eps genes, involved in GLS breakdown and conversion to epithionitriles, respectively, could be associated with the reduction of the level of extra GLS produced under high Se (40 μM). Conversely, in leaves all tested genes related to GLS biosynthesis and breakdown were down-regulated by 40 μM Se, in line with the decreased GLS accumulation, while Eps expression was unaffected (Figure 3). Reduced content of GLS in leaves of plants cultivated in hydroponics was also likely related to the drop of all GLS's amino acid precursors. This effect was neither evident in roots of these plants, nor in leaves and roots of plants grown in soil and exposed to Se, which instead contained higher amount of these amino acids under high Se.

Phenlylalanine, the amino acid precursor of aromatic GLSs, represents also the starting point for the synthesis of phenolic compounds. Its variation in plants cultivated in hydroponics in the presence of Se did not appreciably affect the level of phenolics (Table 5S). However, in plants sprayed with Se, phenylalanine was more accumulated compared to the controls, but a decrease of phenolic compounds was observed, particularly in roots. This result may indicate a possible interference of Se with further steps in phenolic synthesis. In this respect, contrasting results on the effects of Se on phenolic metabolism have been reported in other studies. In *B. rapa*, for instance, the application of SeO_2_ enhanced phenolics accumulation and the expression of genes related to their biosynthesis (Thiruvengadam and Chung, 2015). Similar results were obtained in broccoli (Bachiega et al., 2016) and in tomato (Schiavon et al., 2013) plants fertilized with Se, whereas Robbins et al. (2005) and Tian et al. (2016) found a decrease in the level of phenolics in broccoli.

## Conclusions

Se fertilization of crops can be performed employing different approaches. In this study, we show that Se concentration and the method employed for achieving Se biofortification may differentially alter the production of phytochemicals in plants.

Foliar Se fertilization has been widely used to enhance Se accumulation in the aboveground plant tissues of several crops, but for the first time it is tested for its efficacy to enrich radish edible roots with Se. This method was indeed successful, as Se accumulated in roots, likely as a result of translocation of organic selenocompounds from the leaves. The Se-treated radish roots were not only enhanced with the important anticarcinogenic MSeC but also with the GLS glucoprahanin. No negative effects on amino acid content were observed under Se foliar fertilization, although the level of some phenolic compounds was decreased.

When plants were grown in hydroponics, the elevated capacity of radish to translocate Se from the root to the shoot was evident, a trait useful in other fields (e.g., phytoremediation). Also in this case, MSeC was accumulated in roots, but values were lower compared to those measured in Se-sprayed plants, despite similar values of Se in tissues were measured at the highest Se dosages (20 mg Se per plant and 40 μM Se, in soil and hydroponics, respectively). In hydroponics, the root level of amino acids also decreased at higher Se dosages, and no stimulation of glucoraphanin synthesis was evident. However, phenolic production was not affected.

In conclusion, Se biofortification of radish roots has been proved to be a more efficient method to produce plants with superior health benefits compared to the hydroponic system. The fact that Se assimilation mainly takes place in the chloroplasts would explain the higher capacity of plants sprayed with Se to promptly convert it into Se organic forms that are further delivered to the roots via phloem. It may be performed through different fertilization systems, although Se-foliar fertilization seemed to be more efficient than hydroponics in Se accumulation in the edible part of the plant. The consumption of radishes under the Se dosages applied can be considered safe and useful to fulfill the daily human requirement (10 radishes provided 70 μg Se). Furthermore, plants were enriched in other important nutraceuticals after Se supplementation.

## Author contributions

MS designed and carried out most experiments and wrote the manuscript. MM supervised the work and co-wrote the manuscript. EP co-wrote the manuscript and performed elemental analyses. SD supervised work. CB and AT performed biochemical analyses.

### Conflict of interest statement

The authors declare that the research was conducted in the absence of any commercial or financial relationships that could be construed as a potential conflict of interest.
