# Supplementary material for: Selenium Biofortification in Radish Enhances Nutritional Quality via Accumulation of Methyl-Selenocysteine and Promotion of Transcripts and Metabolites Related to Glucosinolates, Phenolics, and Amino Acids
Source: Front Plant Sci. 2016 Sep 14;7:1371. doi: 10.3389/fpls.2016.01371 (PMC5021693; doi:10.3389/fpls.2016.01371)
Supplement: Supplementary file 1 [file Table1.DOCX]

**Table 1S. Fragmentation pattern of glucosinolates identified in leaves and roots of radish plants, compounds were detected in positive ion mode observing protonated [M+H]^+^, sodium adduct [M+Na]^+^or potassium adduct [M+K]^+^ pseudomolecular ions. Abbreviation DBM-GLS indicate Dimeric-4-mercaptobutyl glucosinolate.**

| **Glucosinolate** | **Fragmentation** | **[M+H]^+^** | | **[M+Na]^+^** | **[M+K]^+^** |
| --- | --- | --- | --- | --- | --- |
|  |  |  |  |  |  |
| **Glucoraphanin** | 216,134 | |  | 378 |  |
| **Glucoraphasatin** |  | |  | 362 | 378 |
| **Glucobrassicin** | 229,219 | |  | 391 |  |
| **Neoglucobrassicin** | 237,160,130 | | 399 | 421 | 437 |
| **DMB-GLS** | 529 | |  |  | 691 |

**Table 2S. Fragmentation pattern of phenolic compounds identified in leaves and roots of radish plants. [M+K]^+^ - potassium adduct pseudomolecular ions; RT – Retention Time**

| **Polyphenol** | **Fragmentation** | **[M+H]^+^** | **RT (min)** |
| --- | --- | --- | --- |
| **Kaempherol-3-glucoside** | 285 | 447 | 4.9 |
| **Kaempherol-7-O-rhamnoside** | 285 | 431 | 6.0 |
| **Caffeic acid** | 135 | 179 | 7.2 |
| **Kaempherol-3-rhamnosil glucoside** | 447,285 | 593 | 7.7 |
| **Kaempherol-3-O-arabinoside-7-O-rhamnoside** | 417,285 | 563 | 7.9 |
| **Kaempherol-3,7-dirhamnoside** | 431,285 | 577 | 8.6 |
| **Coumaric acid** | 119,93 | 163 | 8.7 |
| **Sinapic acid** | 209,179,164,149 | 223 | 9.1 |
| **Ferulic acid** | 179,149,134 | 193 | 9.2 |
| **Feruilmalate** | 193,134 | 309 | 9.2 |
| **Sinapoilmalate** | 223,179,133 | 339 | 12.6 |

**Table 3S.** **Sequences of primers used in qRT-PCR reactions**.

| **Gene name** | **Forward primer 5’-3’** | **Reverse Primer 5’-3’** |
| --- | --- | --- |
| **Sultr1;1** | TGTTCATCACACCGCTCTTC | TGCTGCGTCAATGTCAATAAG |
| **Sultr1;2** | ATGGCTGGATGTCAAACTGC | TCAGAGGAATCACTGCGTTG |
| **Sultr2;1** | TTGGGCTACAAGAAACTCGTC | CTGAAAATCCCGAAAGAAGC |
| **Aps1** | CCCTATCCTTTTGCTTCATCC | GTGCTGCTTCATCCTCCAAC |
| **Aps24** | GAGAAGGTGCTTGAGGATGG | TTGGAGATGGGAAGATGGAG |
| **Myb28** | CCCAAGCAGAAAGGTTTCAA | CCCTAAACTTGGGACTAACAACC |
| **Myr** | GCGAAGAGAACGAACCATTC | GCAACACCGAAGATGAAGTC |
| **Ugt74b1** | GATTCCATCGGCTTACCTTG | CCAAACGAACCAAACGAAAC |
| **Eps** | ACTGGTGTGGGAGAGGTTTG | ATCGGTTCGGTTGTTGGTC |
| **Actin1** | AGCATGAAGATCAAGGTGGTG | CTGACTCATCGTACTCTCCCT |

**Table 4S. Concentration of total selenium (Se), nitrogen (N), sulfur (S), carbon (C) in soil collected from pots where radish plants were cultivated. Data are expressed on a dry weight (DW) basis. Letters following standard deviation (±SD) denote statistical differences among treatments along the same column (p<0.05).**

| **Treatment** | **Se** | **N** | **S** | **C** |
| --- | --- | --- | --- | --- |
|  | **mg kg^-1^ soil DW** | **% (g /100 g^-1^ soil DW)** | | |
| **Control** | 0.32±0.12a | 0.452± 0.012a | 0.174±0.006a | 19.27±1.14ab |
| **Se 5 mg/plant** | 1.81±0.94a | 0.456±0.011a | 0.161±0.015ab | 16.38±1.56b |
| **Se 10 mg/plant** | 1.85±0.56a | 0.442±0.011a | 0.182±0.010a | 22.19±2.21a |
| **Se 20 mg/plant** | 2.75±0.69a | 0.429±0.015a | 0.154±0.008b | 18.74±1.99ab |

**Table 5S. Content of phenolic compounds identified in leaves and roots of radish plants cultivated in hydroponics. Data represent the mean of four biological replicates Different letters along rows indicate significant differences (p< 0.05, ± STD) among treatments.**

| **Polyphenol *(μg kg^-1^ FW)*** | | | **Leaves**  ***Se (mg per plant)*** | | | | |
| --- | --- | --- | --- | --- | --- | --- | --- |
|  |  |  | **0** | **5** | **10** | **20** | **40** |
| **Kaempherol-3-glucoside** | | | 7 ± .3a | 9 ± 6a | 8 ± 2a | 9 ± 2a | 0.008 ± 0.003a |
| **Kaempherol-7-O-rhamnoside** | | | 4 ± 1a | 4 ± 1a | 4 ± 0a | 3 ± 1a | 0.003 ± 0.000a |
| **Caffeic acid** | | | 44 ± 3a | 50 ± 6a | 38 ± 8a | 41 ± 19a | 0.037 ± 0.008a |
| **Kaempherol-3-rhamnosil glucoside** | | | 106 ± 9a | 106 ± 24a | 104 ± 3a | 95 ± 32a | 0.099 ± 0.030a |
| **Kaempherol-3-O-arabinoside-7-O-rhamnoside** | | | 57 ± 6a | 51 ± 7a | 49 ± 4a | 45 ± 14a | 0.042 ± 0.007a |
| **Kaempherol-3,7-dirhamnoside** | | | 90 ± 5a | 96 ± 2a | 98 ± 8a | 94 ± 31a | 0.090 ± 0.018a |
| **Cumaric acid** | | | 184 ± 11a | 190 ± 45a | 168 ± 21a | 169 ± 39a | 0.197 ± 0.047a |
| **Sinapic acid** | | | 15 ± 2a | 20 ± 9a | 39 ± 24a | 20 ± 11a | 0.017 ± 0.012a |
| **Ferulic acid** | | | 153 ± 18a | 159 ± 19a | 143 ± 12a | 143 ± 30a | 0.147 ± 0.010a |
| **Feruilmalate** | | | 73 ± 14a | 87 ± 7a | 72 ± 14a | 67 ± 34a | 0.080 ± 0.036a |
| **Sinapoilmalate** | | | 11 ± 1a | 12 ± 6a | 18 ± 13a | 13 ± 1a | 0.015 ± 0.006a |
| **Total** | | | 744 ± 16ab | 784 ± 25a | 741 ± 16ab | 699 ± 23b | 0.735 ± 0.028ab |
| **Polyphenol *(μg kg^-1^ FW)*** | | | **Roots**  ***Se (mg per plant)*** | | | | |
|  |  | | **0** | **5** | **10** | **20** | **40** |
| **Kaempherol-7-O-ramnoside** | | | 22 ± 4a | 17 ± 5a | 20 ± 4a | 16 ± 5a | 0.022 ± 0.004a |
| **Cumaric acid** | | | 13 ± 5a | 18 ± 5a | 22 ± 7a | 19 ± 3a | 0.015 ± 0.005a |
| **Ferulic acid** | | | 45 ± 14a | 40 ± 9a | 48 ± 9a | 43 ± 7a | 0.038 ± 0.010a |
| **Kaempherol-3-ramnosil glucoside** | | | 9 ± 3a | 8 ± 2a | 9 ± 2a | 7 ± 3a | 0.011 ± 0.003a |
| **Total** | | | 89 ± 7a | 83 ± 6a | 99 ± 6a | 85 ± 5a | 0.086 ± 0.007a |

**Table 6S. Amount of Se (mg per plant) supplied to plants grown in hydroponics.**

| **Se treatment (μM)** | **Corresponding mg Se per plant** |
| --- | --- |
| **5** | **0.4** |
| **10** | **0.8** |
| **20** | **1.6** |
| **40** | **3.2** |
