# Supplementary figures and images for: Selenium Biofortification in Radish Enhances Nutritional Quality via Accumulation of Methyl-Selenocysteine and Promotion of Transcripts and Metabolites Related to Glucosinolates, Phenolics, and Amino Acids
Source: Front Plant Sci. 2016 Sep 14;7:1371. doi: 10.3389/fpls.2016.01371 (PMC5021693; doi:10.3389/fpls.2016.01371)

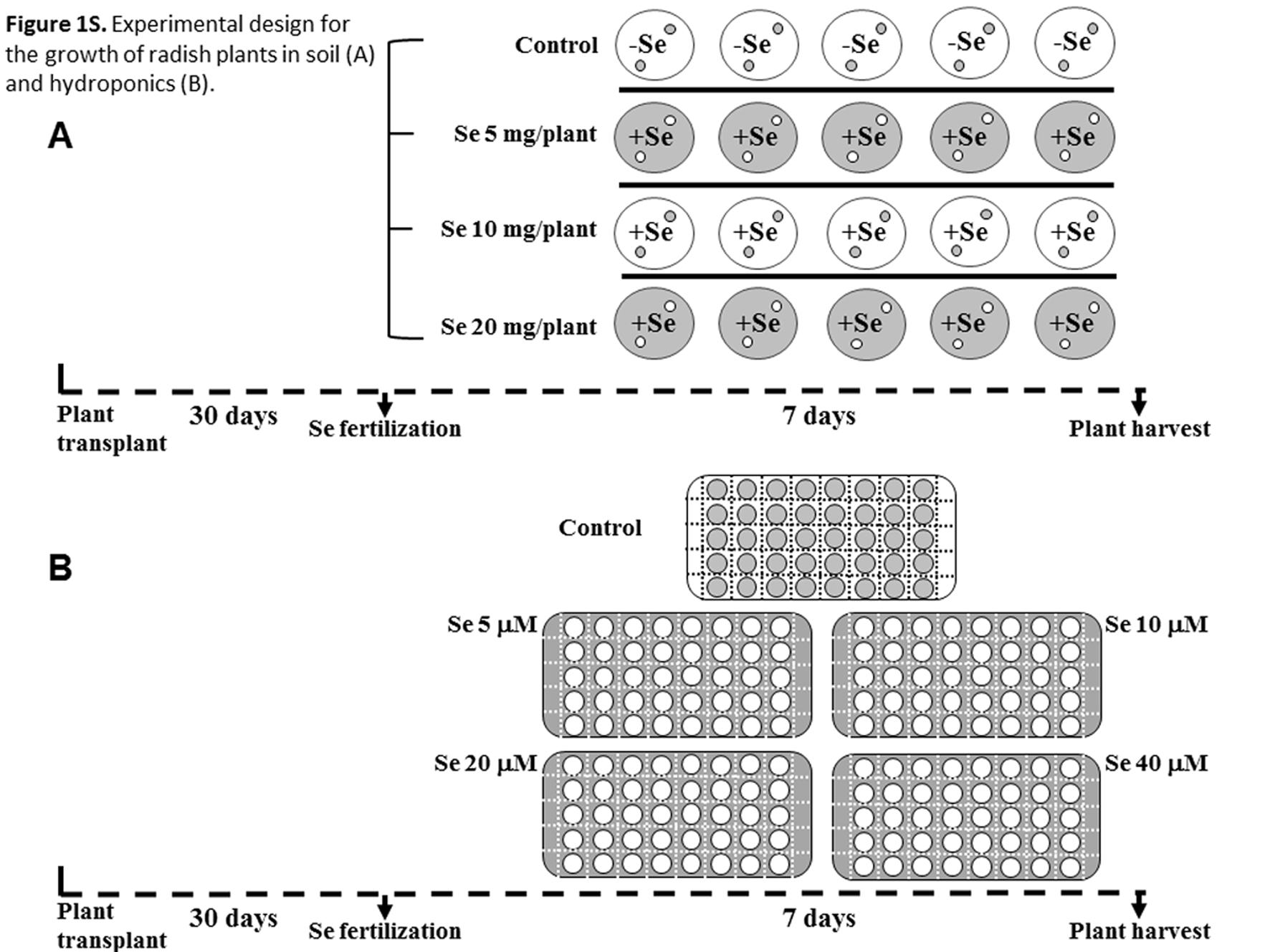

Supplement: Supplementary file 2 [file Image1.TIF]

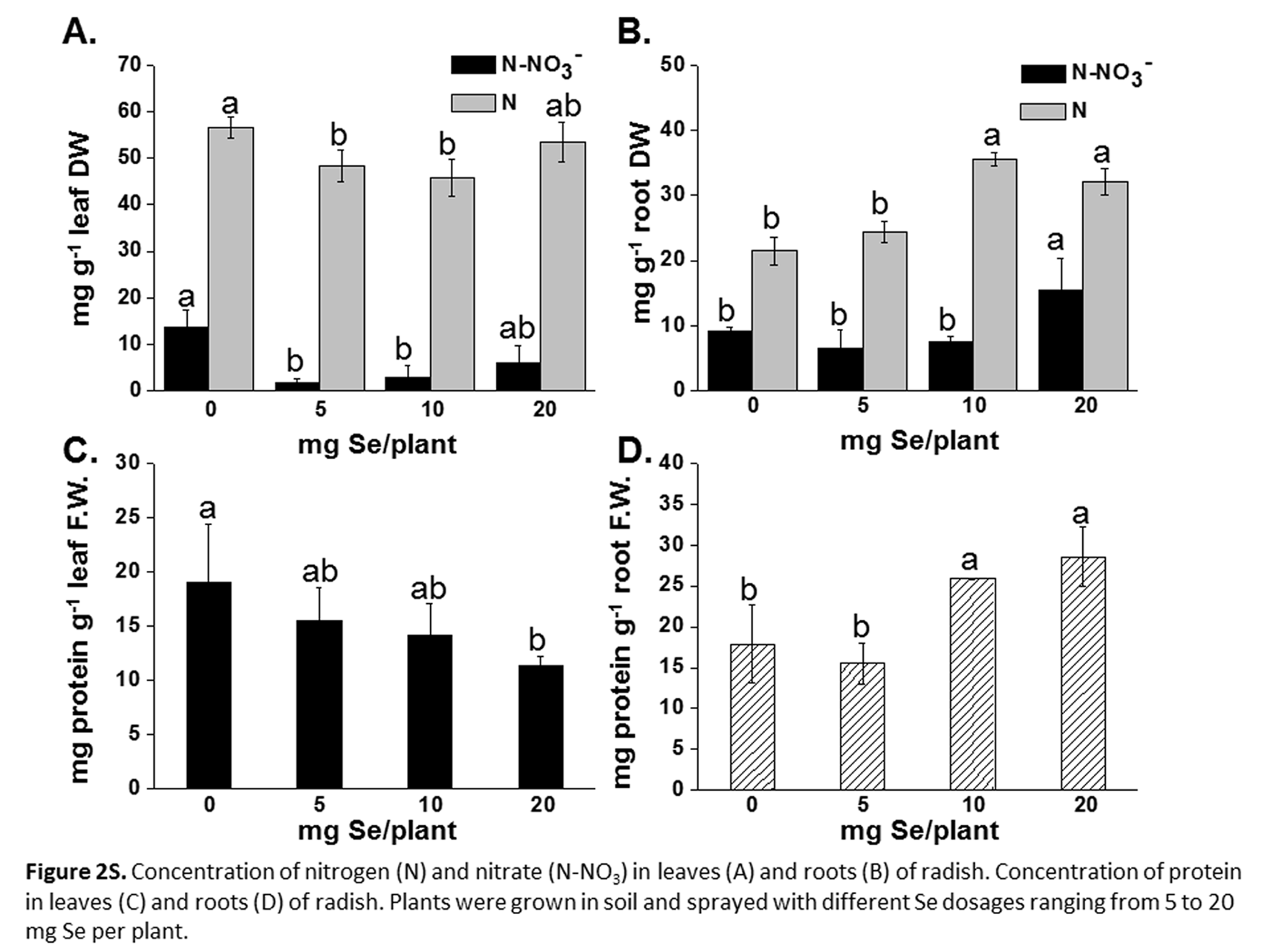

Supplement: Supplementary file 3 [file Image2.TIF]
